# Application of Preoperative Ultrasonography in the Diagnosis of Cervical Lymph Node Metastasis in Thyroid Papillary Carcinoma

**DOI:** 10.3389/fsurg.2022.851657

**Published:** 2022-02-28

**Authors:** Ailong Zhang, Shenglan Wu, Zhenhui You, Wenkai Liu

**Affiliations:** ^1^Department of General Surgery, Fujian Provincial Hospital, Fuzhou, China; ^2^Department of Ultrasonography, Jingzhou City Women and Children Hospital, Jingzhou, China

**Keywords:** lymph node metastasis, contrast-enhanced ultrasonography, ultrasonic examination, thyroid tumor, preoperative

## Abstract

**Background:**

The clinical value and application of preoperative ultrasound contrast in the diagnosis of cervical lymph node metastasis in thyroid papillary carcinoma is investigated.

**Methods:**

In total, 126 cases of thyroid papillary carcinoma were selected, the sensitivity and accuracy of color ultrasound and ultrasound contrast were analyzed by comparing preoperative gray-scale ultrasound, color ultrasound, and ultrasound contrast.

**Results:**

The accuracies of preoperative color ultrasound and ultrasound contrast in detecting lymph node metastasis were 74 and 82%, respectively, and their sensitivities were 80 and 94%, respectively. Lymph node metastasis was significantly more severe when the tumor diameter was >4 cm. The lymphatic metastatic rate of the patients with multifocal papillary carcinoma was 96.4%, whereas the lymphatic metastatic rate of the patients with thyroid gland lesions was 87.7%. The central foci of cervical lymph node metastasis included the following pathological subtypes: diffuse sclerosis type (89.3%, 25/28), high-cell type (72.2%, 8/11), and papillary type (40.0%, 4/10).

**Conclusion:**

Ultrasound contrast is more sensitive than color ultrasound in the diagnosis of cervical lymph node metastasis. Primary lesions ≥4 cm, lesion involvement, outer membrane, and high-risk pathologic subtypes and lesions were considered as the criteria for ultrasound contrast application.

## Introduction

Papillary thyroid carcinoma is the most common type of thyroid malignancy and accounts for over 80%. Most patients with this malignancy experience slow disease progress and have a good prognosis, but 30–80% of them might experience potential cervical lymph node metastasis (1, 2). However, whether cervical lymph node metastasis affects the prognosis of papillary thyroid carcinoma remains controversial. Some factors might influence the lymph node metastasis of papillary thyroid carcinoma. It was reported that vascular invasion, multifocality, large tumor diameter, young age, and male sex were positively correlated with central lymph node metastasis in the cervical region in papillary thyroid carcinoma patients (3). In papillary thyroid cancer patients, Hashimoto's thyroiditis has a protective effect on central lymph node metastasis and a risk effect on lateral lymph node metastasis (4).

The standard surgical treatment for this disease is cervical lymphatic dissection, but excessive or insufficient neck cleaning is common in clinics because of the difficulty in accurately diagnosing cervical lymph node metastasis before operation (1). Lymph node biopsy is a pathological analytical method with high diagnostic accuracy, which is yet to be widely applied in China (5). Although ultrasound, CT, and MRI have significantly increased the diagnostic rate for this disease, several deficiencies still remain (6). Thus, ultrasound contrast has shown potential application value in diagnosing diseases.

In this study, we analyzed the factors influencing the sensitivity of this technique and discussed the value and application of preoperative ultrasonography in diagnosing cervical lymph node metastasis from thyroid papillary carcinoma patients. The diagnostic sensitivity and accuracy of color ultrasound and ultrasound contrast for thyroid papillary carcinoma patients were analyzed.

## Materials and Methods

### General Materials

In total, 126 thyroid papillary carcinoma cases were enrolled in our department from November 2015 to November 2020. This study was approved by the Institutional Animal Care and Use Committee (approval number: 2015-36). Oral informed consent was obtained from subjects. All experiment procedures are in accord with the Declaration of Helsinki. These cases included 28 thyroid papillary carcinoma cases, but clinical palpation or imaging examination revealed that these cases were enlarged with neck lymph nodes. Grayscale ultrasound, color ultrasound, and ultrasound contrast were conducted before operation. The patients included 31 men and 95 women. The ages ranged from 19 to 77 years with a median age of 46 years. Among the cases, 68 cases were ≥45 years and 58 cases were <45 years. The sizes of their thyroid nodules ranged from 4 to 42 mm and the median was 20 mm. The following conditions were observed: unilateral carcinoma in 98 cases, bilateral carcinoma in 17 cases, and multicentral carcinoma in 11 cases.

### Ultrasonic Examination

Philips ultrasonic diagnostic equipment with a frequency range of 8–13 MHz, a mechanical index (MZ) of 0.05–0.08, and a compressed range of 33–35, dynamic shear rheometer (DSR) (Middle) was used in this study. An ultrasound contrast agent (Sonovue; Bracco, Milan, Italy) was combined with 5 ml saline and shaken for 30 s. A 2 × 10^8^/mm^3^ microbubble suspension was formed by intravenously injecting each contrast agent into the elbow before the vein with a 2.4 ml static push and 5 ml saline was used for flushing tubes. At the same time, the timer was pressed and the characteristics of the lymph node perfusion and the intensity changes in echo were observed using a double-image model. Automatic tracking radiography analysis software (ACQ) was used. The morphological characteristics, aspect ratio, internal echo, and the lymphatic gate of the lymph nodes were observed through conventional 2D ultrasonography. The pattern of vascular distribution in the lymph nodes was examined with color ultrasound. Contrast-enhanced ultrasound showed the characteristics of “uneven enhancement” and “fast forward and slow backward” that indicated cancer metastasis. All images and other dynamic images were stored in this machine or in a universal serial bus (USB) disk for retrospective analysis. Two individuals were designated to analyze the image data. In cases of inconsistencies in qualitative data, they agreed to include data after consultation.

### Operation Method

In routine neck incision or original incision, the primary lesion was limited to the side of lateral and isthmic resections. The primary lesion invaded by the membrane or involved two lobes, routine sending rapid pathological examination report thyroid papillary carcinoma and the incision was extended after the ipsilateral sternocleidomastoid muscle was freed in the middle and lower parts of the posterior margin. The initial lesion was located on the upper lobe of the glandular leaves the first sweep of III, IV, and VI regionsof the lymphoid adipose tissue. The primary lesion was located on the anterior pituitary gland, the first sweep of the VI area of the lymphoid adipose tissue. The results of the frozen pathological examination were transported for detecting cancer metastasis and the entire neck sweep of the lateral area. The postoperative patients with thyroid papillary carcinoma were treated with standard side necks II–V zone sweeping.

### Pathological Examination

The pathological report of each paraffin section was used as the diagnostic criterion for patients. After surgery, the paraffin was sent to the pathology department, and according to II–V groups of lymph node packet, the size of the tumor was recorded, and invasion of the thyroid gland membrane was determined. Neck lymph node area, sweeping amount, metastasis number, lymph node shape, size, aspect ratio, and calcification were determined. Lymph node micrometastasis was continuously sliced and examined by two pathological experts. For the patients with thyroid papillary carcinoma, the paraffin sections of the thyroid lesion were reviewed. The sections were then classified into the following pathological subtypes: large follicular type (1 case), papillary microcancer (10 cases), follicular type (10 cases), eosinophilic cell type (7 cases), solid variant type (9 cases), fascia type (9 cases), hyaline cell type (10 cases), high cell type (11 cases), diffuse follicular type (3 cases), columnar cell type (11 cases), diffuse sclerosis type (28 cases), and typical papillary carcinoma (25 cases).

### Statistical Analysis

Data were statistically analyzed using Statistical Product and Service Solutions (SPSS) 18.0 (SPSS Inc., Chicago, IL, USA). The counted data for the different groups were compared with *X*^2^ test, and a multivariate analysis was conducted through logistic regression analysis. *p* < 0.05 was considered statistically significant.

## Results

### General Material Analysis

In total, 95 patients had lateral cervical lymph node metastasis (total metastatic rate: 75.4%), 89 patients had central region lymph node metastasis (total metastatic rate: 70.6%), 47 patients had group II lymph node metastasis (total metastatic rate: 37.3%), 61 patients had group III lymph node metastasis (total metastatic rate: 48.4%), 50 patients had group IV lymph node metastasis (total metastatic rate: 39.7%), and 11 patients had group V lymph node metastasis (total metastatic rate: 8.7%).

No significant difference was observed as for the age and gender of patients with cervical lymph node metastasis of papillary thyroid carcinoma. Lymph node metastasis was significantly more severe if tumor diameter was >4 cm (*X*^2^ = 8.3, *p* < 0.05). The lymphatic metastatic rate of multiple central papillary carcinomas reached 96.4% (27/28). The lymph node metastatic rate was 87.7% (64/73) in the patients with thyroid gland membrane invasion, whereas the cervical lymph node metastasis was 58.5% (31/53) in the patients with the thyroid gland membrane, and the difference was significant. The primary lesion was located on the surface of the gland leaves in zones II–IV lymph node metastases (39, 49.5, and 41.9%, respectively), but the primary lesion was located at the bottom of gland leaves in the main VI regional lymph node metastasis (62.8%). The central lesions of cervical lymph node metastasis were classified under the following pathologically subtypes: diffuse sclerosis type (89.3%, 25/28) and high cell type (72.2%, 8/11). These values are significantly higher than those of the papillary micro-carcinoma type (40%, 4/10; Tables 1, 2).

**Table 1:** Distribution of thyroid lesion sites and regional lymph nodes (cases).

**Thyroid lesions**	**II**	**III**	**IV**	**V**	**VI**
Upper level	23	29	44	2	10
Medium level	18	23	20	3	13
Low level	6	9	6	7	66
Total	47	61	50	11	89

**Table 2:** Pathological subtype invasion and metastasis of thyroid papillary carcinoma (cases).

**Pathological subtype**		**Lymph node metastasis in the cervical area**	**Invasion of the outer glands**
Diffuse sclerosis type	28	25	28
High cell type	11	8	11
Diffuse follicular type	3	3	2
Columnar cell type	3	2	3
Follicular type	10	7	3
Solid variant type	9	7	3
Fascia type	9	6	3
Hyaline cell type	10	7	4
Eosinophilic cell type	7	6	3
Typical papillary carcinoma	25	19	12
Papillary microcancer	10	4	1
Large follicular type	1	1	0
Total	126	95	73

Preoperative color ultrasound and ultrasound contrast detected 74 and 82% patients with total lymph node metastasis, respectively (*X*^2^ = 16.457, *p* = 0.158). The sensitivity of ultrasound contrast was 94%, the positive predictive value was 83.9%, and the negative predictive value was 70% (Table 3).

**Table 3:** Diagnosis by color ultrasound and contrast-enhanced ultrasonography.

	**Sensitivity**	**Positive predictive value**	**Negative predictive value**	**Accuracy**
Color ultrasound	(76/95) 80%	(76/90) 84.4%	(17/36) 47.2%	(93/126) 74%
Ultrasound contrast	(88/95) 94%	(89/106) 83.9%	(14/20) 70%	(103/126) 82%
*P*-value	0.102	0.974	0.449	0.158

### Analysis and Comparison of Sensitivity Between Color Ultrasound and Contrast-Enhanced Ultrasonography in Diagnosing Cervical Lymph Node Metastasis

We examined the positive rate of ultrasonography and the total number of regional lymph node metastasis in terms of diagnostic sensitivity. The sensitivity of color ultrasound in detecting cervical lymph node metastasis was 80%. A total of 13 cases of cervical lymph node metastasis were detected through color ultrasound and contrast echocardiography. The clinical and histological subtypes of the patients with lymph node metastasis were compared through color ultrasonography, and the results revealed that 10.3% of the patients were diagnosed with small calcification (68%). The sensitivity of contrast-enhanced ultrasonography used in the detection of cervical lymph node metastasis was 94% (*X*^2^ = 2.5002, *p* = 0.102). Such a high sensitivity might be associated with the unique microcirculation of lymph node metastasis. Logistic regression analysis showed that lymph node calcification was correlated with color ultrasonic diagnostic sensitivity in the following parameters: age, tumor size, histologic subtype, lymph node calcification, aspect ratio, necrosis, and blood flow signal richness [odds ratio (OR) = 8.723, *p* = 0.007]. The abundance of blood flow signals was correlated with the diagnostic sensitivity of ultrasound angiography (OR = 3.481, *p* = 0.029). Color ultrasound was significantly different from contrast-enhanced ultrasonography in terms of the diagnosis of lymph node metastasis.

## Discussion

Ultrasonography is the most widely used method for the preoperative diagnosis of thyroid carcinoma and cervical lymph node metastasis (7). CT, MRI, and 2-deoxy-2-[fluorine-18]fluoro-D-glucose (18-FDG) PET are not recommended by the latest domestic and international guidelines for routine examination (1). Ultrasonography is the first method utilized for the evaluation of lymph node metastasis in thyroid carcinoma and cervical region and an accuracy rate of 72% is reported in the literature (8, 9). In this study, the accuracy of color ultrasound in the diagnosis of cervical lymph nodes was 74%, which is consistent with the reported value. Our results also found that ultrasound contrast was accurate (82%) and highly sensitive, suggesting that ultrasound contrast has an enhanced clinical application value.

**Figure 1:**
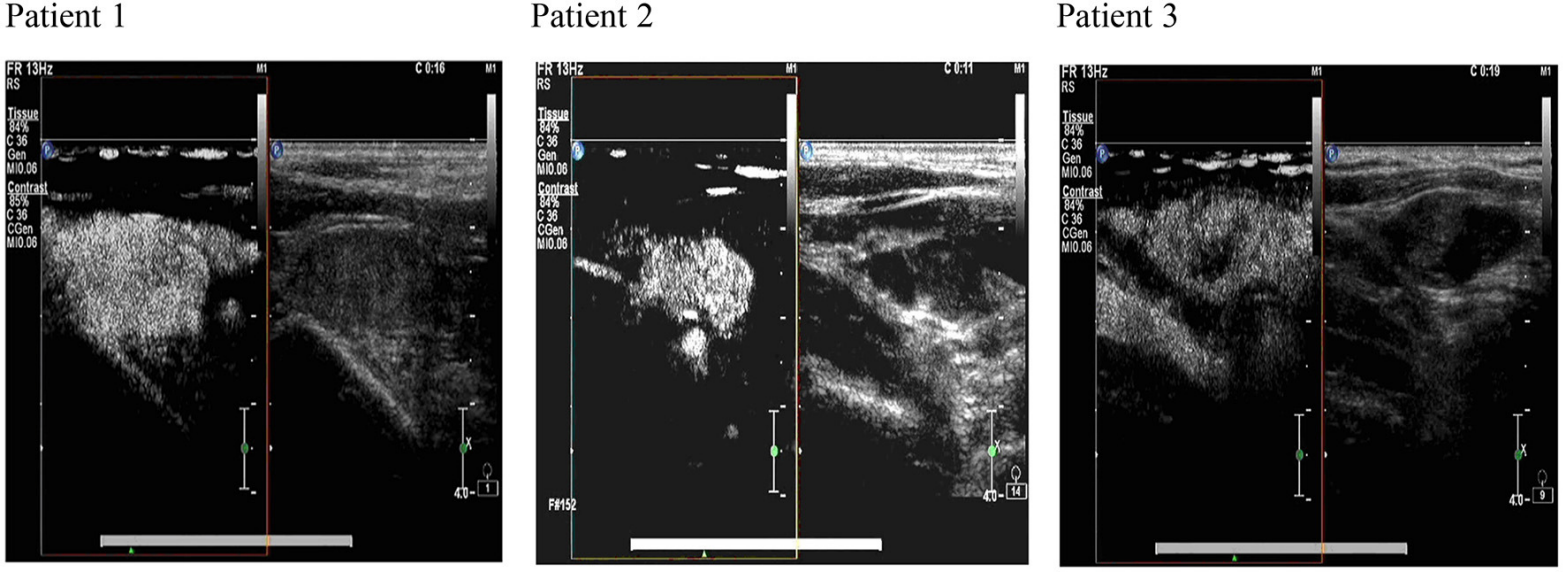
The ultrasonography image of cervical lymph nodes in comparison with ultrasound contrast and color ultrasound images. The images produced by color ultrasound are used for the evaluation of the nature of lymph node lesions. The characteristics of “uneven enhancement” and “fast forward and slow backward” are observed after ultrasound contrast. Postoperative pathological analysis confirms lymph node metastasis.

Some studies have shown that the calcification of lymph nodes, the delineation of the medulla, an aspect ratio of >1, the disappearance of the lymphatic gate, central liquefaction necrosis, and peripheral enhancement are the criteria for determining lymph node metastasis (10). However, this standard has limitations. Small lymph nodes with normal morphological sizes are common, whereas lymph node calcification, fluid necrosis, and peripheral enhancement do not rarely indicate metastasis (11). Moreover, cervical lymph node inflammation is also visible (12). Some researchers found that the lymph node metastatic rate of thyroid papillary carcinoma (CN0) was 60% when the cervical lymph node was swept during operation (13). In other reports, patients with chronic lymphocytic thyroiditis often exhibit neck lymph node enlargement, lymph node echo heterogeneity reduction, and lymphatic door disappearance (14, 15). We used ultrasound contrast technology to address these deficiencies. The diagnosis of positive lymph nodes can reveal not only their morphological characteristics but also the “uneven reinforcement” and “fast forward slow backward” behavior of local blood supply in ultrasound contrast. In typical cases, as shown in Figure 1, the morphological structure of lymph nodes in grayscale and color ultrasound images is generally normal. “Uneven enhancement” and “fast forward slow backward” are only observed after ultrasound contrasts and are consistent with the pathological examination results. However, for the regional lymph nodes in grayscale, the color ultrasound performance of typical audio-visual transfer, especially low degree of calcification, the aspect ratio of >1, the abundance of blood flow signal, and the overall accuracy of ultrasound contrast was 86% compared with that of the other technique (84%), but the difference between these techniques was not significant. This result suggested that ultrasound contrast was advantageous over other ultrasound methods in terms of the diagnosis of patients with capability in diagnosing suspected lymph node metastasis.

At present, the pathogenesis of cervical lymph node metastasis in papillary thyroid carcinoma is not established. Some studies reported that thyroid papillary carcinoma invades the membrane or the surrounding tissues or organs, and the metastatic rate in cervical lymph nodes was 46.9% (13). In the present study, the patients with membranous invasion were more prone to lymph node metastasis than those without invasion, and the normal grayscale and color ultrasound results were not transferred. The lesions were located on the upper-medium lobe of the gland mainly in the IIA, III, and IV regional lymph node metastases (39, 49.5, and 41.9%, respectively). The primary lesion was found at the low level mainly in the zone VI regional lymph node metastasis (62.8%). Cancer lesions detected at the upper-medium level are likely to be transferred to zones II and III. Most of the lower-level lesions are transferred to the VI area, which should be targeted to “focus” on the key areas of lymph nodes.

In the present study, the pathological subtypes of primary thyroid tumor were combined with ultrasound contrast. The lymph node metastatic rates were 89.3, 72.2, and 66.7% in the cervical regions with diffuse-type, high-cell-type, and columnar-cell-type pathologies. The cervical lymph node metastatic rate was 96.4%. Under the following conditions, ultrasound contrast examination should be actively performed: (1) maximum diameter of the primary lesion ≥40 mm; (2) lesions are involved in the membrane or invading the surrounding tissues or organs; (3) diffuse type, high cell type, columnar cell type, and other high-risk groups; and (4) multicentric primary lesion. The reoperation rate should be reduced, and a precise direction of neck-selective area sweeping should be followed.

The study has several limitations. This research is a retrospective analysis and may be limited by potential selective bias. Moreover, the research is limited by case group, the absence of benign thyroid tumor, and the presence of cervical lymph node enlargement of the case for comparison. The diagnostic specificity of ultrasound contrast was also difficult to compare. Further studies should be performed to achieve objective observations.

## Conclusion

In conclusion, the study showed that ultrasound contrast is more sensitive and accurate than color ultrasound in the diagnosis of cervical lymph node metastasis, especially recessive lymph node metastasis, of thyroid papillary carcinoma. Ultrasound contrast also has several of the following advantages. For example, it does not cause trauma, emit radiation, and elicit toxic effects. Furthermore, it requires simple operation and provides good clinical value.

## Data Availability Statement

The original contributions presented in the study are included in the article/supplementary material, further inquiries can be directed to the corresponding author.

## Ethics Statement

The studies involving human participants were reviewed and approved by the Ethics Committee of Fujian Provincial Hospital. The patients/participants provided their written informed consent to participate in this study.

## Author Contributions

AZ and SW designed the study. SW and ZY collected the data. ZY and WL analyzed the data. AZ prepared the manuscript. All authors read and approved the final manuscript.

## Funding

This work was supported by the Science Foundation of the Fujian Province, China (grant no. 2016J1494).

## Conflict of Interest

The authors declare that the research was conducted in the absence of any commercial or financial relationships that could be construed as a potential conflict of interest.

## Publisher's Note

All claims expressed in this article are solely those of the authors and do not necessarily represent those of their affiliated organizations, or those of the publisher, the editors and the reviewers. Any product that may be evaluated in this article, or claim that may be made by its manufacturer, is not guaranteed or endorsed by the publisher.
